# Chromosome territory repositioning induced by PHA-activation of lymphocytes: A 2D and 3D appraisal

**DOI:** 10.1186/s13039-015-0146-3

**Published:** 2015-07-03

**Authors:** Dimitrios Ioannou, Lakshmi Kandukuri, Joe Leigh Simpson, Helen Ghislaine Tempest

**Affiliations:** Department of Human and Molecular Genetics, Herbert Wertheim College of Medicine, Florida International University, Miami, FL 33199 USA; Present address Centre for Cellular and Molecular Biology (CCMB), Council of Scientific and Industrial Research (CSIR) Uppal Road, Hyderabad, 500 007 India; Biomolecular Sciences Institute, Florida International University, Miami, FL 33199 USA

**Keywords:** Genome organization, Chromosome territories, Phytohaemagglutinin, Repositioning

## Abstract

**Background:**

Genomes and by extension chromosome territories (CTs) in a variety of organisms exhibit nonrandom organization within interphase nuclei. CTs are susceptible to movement upon induction by a variety of stimuli, including: cell differentiation, growth factors, genotoxic agents, proliferating status, and stimulants that induce novel transcription profiles. These findings suggest nuclear architecture can undergo reorganization, providing support for a functional significance of CT organization. The effect of the initiation of transcription on global scale chromatin architecture has been underexplored. This study investigates the organization of all 24 human chromosomes in lymphocytes from two individuals in resting and phytohaemagglutinin activated lymphocytes using 2D and 3D approaches.

**Results:**

The radial organization of CTs in lymphocytes in both resting and activated lymphocytes follows a gene-density pattern. However, CT organization in activated nuclei appears less constrained exhibiting a more random organization. We report differences in the spatial relationship between homologous and heterologous CTs in activated nuclei. In addition, a reproducible radial hierarchy of CTs was identified and evidence of a CT repositioning was observed in activated nuclei using both 2D and 3D approaches.

**Conclusions:**

Alterations between resting and activated lymphocytes could be adaptation of CTs to the new transcription profile and possibly the formation of new neighborhoods of interest or interaction of CTs with nuclear landmarks. The increased distances between homologous and heterologous CTs in activated lymphocytes could be a reflection of a defensive mechanism to reduce potential interaction to prevent any structural chromosome abnormalities (e.g. translocations) as a result of DNA damage that increases during lymphocyte activation.

## Background

Genomes contain the blueprints of life and are organized in-vivo as chromosomes. Nonetheless, our understanding of the spatial organization of genomes and its function has received little attention compared with the vast body of knowledge of the majority of other cellular structures. Pioneering visualization experiments during the 1980s using fluorescence in situ hybridization (FISH) demonstrated that chromosomes were not randomly organized in mammalian cells, occupying distinct positions known as chromosome territories (CTs) [1–6]. These CTs are roughly spherical in shape and 2–4 μm in diameter [7]. Analyses using 3C technology have confirmed nonrandom organization of the genome with current evidence supporting a fractal globule organization of chromatin at all levels, from CTs to chromosome arm and band domains to megabase-sized chromatin built up from a series of spatially separated 100 kbp chromatin domains [8]. Certain biophysical properties of the fractal globule (reviewed in [9]) further support this appealing model for chromatin organization.

Observations in different cell types and organisms identified proximity patterns of chromosomes, leading to the proposal of two models (gene density and chromosome size) for the radial arrangement of CTs. The gene density model proposes that gene-rich CTs and gene-dense subchromosomal regions are located toward the nuclear interior, with gene-poor regions located toward the nuclear periphery [8, 10, 11]. This model originated from observations in proliferating lymphoblasts and fibroblasts and can be seen in primates, old world monkey, rodents, birds (excluding chicken) and cattle [6, 12–14]. The chromosome size model originated from observations in flat ellipsoid fibroblasts, quiescent, and senescent cells proposing localization of larger chromosomes toward the nuclear periphery and smaller chromosomes toward the nuclear interior [15–17]. It is likely that these two models are not mutually exclusive, with radial CT organization depending on the proliferating status of the cells, the chromosome itself and the neighborhood surrounding it, which could play a vital role in regulating cell-type specific gene expression [18].

The above correlative observations of CTs and gene positioning have established the concept of nonrandom organization and highlight emphatically the question of whether there is a link between position and genome function. Evidence to support the hypothesis for a link between position and function is provided from studies of cellular differentiation processes. Examples include the repositioning of the immunoglobulin gene cluster, the Mash1 locus during neural induction [19, 20], the HoxB1 gene in mouse embryos [21], the repositioning of adipogenesis genes during porcine mesenchymal stem cell adipogenesis [22] and sex chromosome movement during porcine spermatogenesis [23]. These studies correlate gene repositioning (to a more internal localization) upon transcriptional activation (with the exception of the sex chromosome movement in pigs). However, internal positioning and activation of genes seems to be an oversimplification since biallelicaly expressed genes, RNA polymerase II sites and heterochromatin can be found throughout the nucleus [21, 24]. Further evidence of gene repositioning, relative to their respective CTs or to other nuclear structures such as transcription factories and splicing speckles also reveals a correlation with transcriptional activity [8]. This evidence comes from studies using active and inactive genes [25], immune-FISH approaches in Ikaros protein [26], 3D positional experiments of adenine nucleotide translocase genes on the X chromosome [27], immediate activation of Myc proto-oncogene in mouse B lymphocytes [28] and the distance between promyelotic leukemia loci and nuclear bodies that seems to correlate with transcriptional activity [29]. More recent evidence from 3C and 4C conformation experiments shows an association of actively transcribed Hox genes in a cluster compared to silent genes that are located in a different region and form part of the active cluster once they are activated [30]. Another emerging feature of genome organization that may play a major role in the control of gene expression is the intraorganization of chromosomes within the CTs in 3D space. This refers to loops that are being formed in order for regions of chromosomes to interact in *cis* (e.g. locus control region of the β-globin gene which acts as an enhancer of the β-globin genes), or *trans* (e.g. in mouse erythroid cells) [7]. The most prominent example of this intraorganization of CTs occurs during X chromosome inactivation in which the ncRNA Xist silences one of the X chromosomes in females, with only a handful of genes escaping inactivation. The X chromosome is inactivated by being condensed into a compact structure (Barr body) that is associated with the nuclear periphery. Following silencing, a repressive nuclear compartment forms that does not affect RNA polymerase II and transcription factors. Genes that are not expressed are “pulled” down into this repressive compartment, rendering themselves inaccessible to transcriptional machinery, whereas the few genes that escape inactivation and are expressed, loop out from this compartment [31]. The aforementioned examples highlight the relationship between the nonrandom organization of chromosomes and gene expression.

Another emerging aspect of the complex nature of genome organization arises from experiments in which the local environment changes by inducing differentiation or transcription through addition or removal of growth factors in cells that exist in culture, or through in-vitro exposure to genotoxic agents. Such experiments could be important in understanding the changes in genome organization as cells divide, age or reach the end of their replicative status. Evidence from primary fibroblasts that entered quiescence (after a 7 day serum starvation) depicts alterations in the topology of CTs including 13, 18 and 10 [16]. With chromosomes 13 and 18 exhibiting a movement toward the nuclear interior and chromosome 10 moving from an intermediate position to a more peripheral one [32]. A more detailed look at the gene expression of ten genes on chromosome 10 following this movement showed that two genes were down-regulated and five were up-regulated when CT 10 was in the periphery providing small scale evidence that the nuclear periphery is not solely associated with gene silencing [32]. Interestingly, the same group measured timing of the relocation of chromosomes after elimination of serum and remarkably CTs relocated within 15 min, highlighting need for energy for this type of repositioning and implicating myosin 1β as the mediator for this relocation [32]. In terms of cells in senescence, evidence from dermal fibroblasts demonstrates that CTs orient themselves following a size-related, rather than gene density related model [33]. These changes are another manifestation of the modification of nuclear architecture during these specific cellular stages that could lead to changes in the transcriptional status of the cells. Nuclear architecture is also subject to alterations when stimuli initiate cellular growth, transcriptional activation or induce DNA damage in vitro. The most prominent example for the former comes from pig mesenchymal stem cells when adipogenic growth factors added in culture give rise to committed pre-adipocyte cells. Six genes involved in the adipogenesis pathway repositioned to a more interior location after 14 days of treatment, correlated with up-regulation. The GATA2 gene moved from a peripheral to an interior location (day 7 – up-regulation) and back to a peripheral location (day 14 – down-regulation) [32]. Recently, we reported reproducible events of CT repositioning in human lymphocytes following in-vitro exposure to genotoxic agents, hydrogen peroxide and UVB [20]. Differences were also reported in CT repositioning between the two genotoxic agents most likely represents differences in mobility and/or decondensation of CTs as a result of differences in the DNA damage induced, chromatin regions targeted and different repair mechanisms [20]. With regards to stimulating a different transcriptional profile a single study has provided evidence of CT repositioning in human lymphocytes following activation of lymphocytes using phytohaemagglutinin (PHA). PHA is a plant mitogen that induces the proliferation of mammalian lymphocytes and creates a cascade of biochemical events that activates resting lymphocytes, which results in large scale decondensation of chromatin, increases in nuclear size and leads to a distinct transcriptional profile [34]. Branco et al. [34] investigated the positioning of 11 CTs in resting human lymphocytes (-PHA) and activated human lymphocytes (+PHA) from a single female subject [34]. The findings showed some intraorganization of CTs with chromosomes 1 and 3 moving more peripherally and chromosome 21 being more centrally located in activated cells [34]. The differences between the two states were attributed to the nuclear expansion as a result of lymphocyte activation by PHA, and to the different transcriptional program [34]. Another important finding was the observation that CT intermingling was lower in activated lymphocytes. This finding was proposed to be a potential protective mechanism to prevent chromosome translocations or risk from DNA damage due to the controlled cell death program that occurs during T lymphocyte activation [34].

Altered transcription profiles induces changes in the topology of CTs; therefore, our study will expand on the previous PHA study [34] to investigate whether the organization of all 24 CTs differs between resting (-PHA) and activated (+PHA) human lymphocytes. Lymphocytes were thus obtained from two volunteers (1 male, 1 female) and CT organization for all 24 CTs was assessed utilizing both 2D and 3D approaches. The purpose of this study was to assess the topology of all 24 chromosomes in resting and transcriptionally activated lymphocytes and where possible compare 2D and 3D approaches. This study specifically investigates: i) random/nonrandom CT organization (2D); ii) spatial relationship between homologous CTs (intraprobe) and heterologous CT pairs (interprobe) (3D); iii) hierarchical organization of CTs from the nuclear interior toward the nuclear periphery (2D and 3D); and iv) CT repositioning in PHA activated lymphocytes (2D and 3D).

Findings in this study suggest that CT organization in lymphocytes is reproducible among subjects and follows a gene density pattern as identified by both 2D and 3D approaches. In addition, a number of alterations in CT organization was observed in activated lymphocytes compared to resting lymphocytes including: i) a less constrained CT organization (more random organization); ii) differences in the spatial organization of homologous and heterologous CTs; iii) small differences in the radial CT hierarchy; and iv) evidence of CT repositioning for a handful of CTs. When possible, comparisons between 2D and 3D approaches revealed largely similar results.

## Results

Although both 2D and 3D approaches can be used to address the same problems, they utilize different approaches allowing different perspectives to be studied. Specifically, the 2D approach examines the radial distribution of the entire CT within a flattened 2D nucleus. The 3D approach utilizes the geometrical center of the CT (single point) and performs measurements in microns to the nearest nuclear edge or geometrical center of another CT, providing a physical location or distance within a 3D nucleus.

### 2D radial chromosome territory organization in resting and activated lymphocytes

**Fig. 1:**
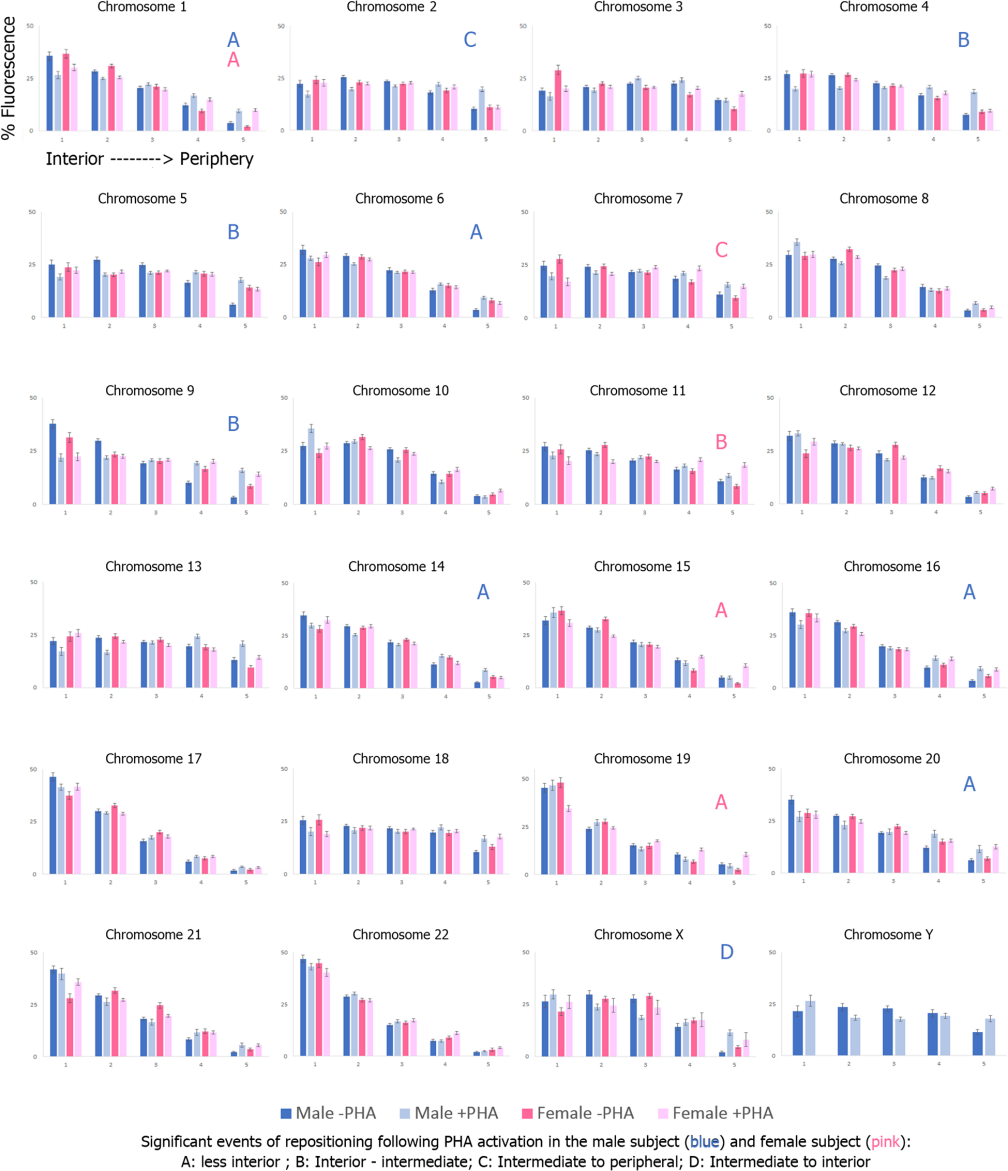
Radial organization for all 24 CTs in resting and activated lymphocytes. Displays the radial distribution for all 24 CTs (chromosomes 1–22, X and Y) in both enrolled subjects for resting (-PHA) and activated (+PHA) lymphocytes. The X-axis for all histograms represents each of the five rings of equal area (1–5) moving from the nuclear interior toward the nuclear periphery (left to right). Each ring includes the data for the resting lymphocytes (dark blue and dark pink) and the activated lymphocytes (light blue and light pink) in the male and female subject, respectively. The Y-axis for all histograms represents the proportion of fluorescence (%) for each CT within each of the five rings. Error bars represent the standard error of the mean (SEM). Significant events of repositioning in activated lymphocytes compared to resting are denoted by letters (**a–d**) in the top right corner of each histogram, blue and pink letters correspond to repositioning events in the male and female subject, respectively. Each letter corresponds to the type or repositioning movement observed based on the radial distribution (see figure key for more details)

The 2D organization of CTs within lymphocytes was assessed using previously published and validated methodologies that divide the interphase nucleus into five equal areas based on DAPI fluorescence intensity, and measures the fluorescence distribution of each CT across the five equal areas [10, 20, 26, 35]. This provides the ability to determine if a CT is equally distributed across the nucleus (random organization), or demonstrates a preferential nuclear localization (nonrandom organization). A total of 9400 cells were captured and analyzed to assess the radial nuclear organization for all 24 human chromosomes in resting (-PHA) and activated (+PHA) lymphocytes. The CT distribution and random/nonrandom status of all 24 CTs is presented in Fig. 1 and Table 1, respectively, for both subjects and in both resting and activated lymphocytes. In resting lymphocytes, the vast majority of CTs occupied nonrandom positions (*p* <0.05), 22 CTs (91.67 %) and 21 CTs (91.30 %), in the male and female subject respectively (Table 1). Nineteen CTs demonstrated nonrandom organization in both subjects (CT Y excluded). In activated lymphocytes a different picture of nuclear organization emerges, with CTs demonstrating a more random organization compared to resting lymphocytes. In activated lymphocytes, 14 CTs (58.33 %) and 16 CTs (69.56 %) occupied a nonrandom organization in the male and female subject, respectively (Table 1). CTs that demonstrated a random organization in resting lymphocytes in the male and female subject (CTs 3 and 13; and CTs 5 and 18, respectively) retained their random organization in activated lymphocytes. In the activated lymphocytes, an additional 8 CTs in the male subject and 5 CTs in the female subject were found to be randomly organized. Of the CTs that were randomly organized in the activated lymphocytes seven were common in both subjects (CTs 3, 5, 7, 9, 11, 13 and 18), whereas CTs 2, 4 and Y were randomly organized only in the male subject.

**Table 1:** Random/nonrandom organization status of all 24 CTs in 2D resting and activated lymphocytes

CT	Male	Female	Male	Female
	-PHA	-PHA	+PHA	+PHA
1	NR	NR	NR	NR
2	NR	NR		NR
3		NR		
4	NR	NR		NR
5	NR			
6	NR	NR	NR	NR
7	NR	NR		
8	NR	NR	NR	NR
9	NR	NR		
10	NR	NR	NR	NR
11	NR	NR		
12	NR	NR	NR	NR
13		NR		
14	NR	NR	NR	NR
15	NR	NR	NR	NR
16	NR	NR	NR	NR
17	NR	NR	NR	NR
18	NR			
19	NR	NR	NR	NR
20	NR	NR	NR	NR
21	NR	NR	NR	NR
22	NR	NR	NR	NR
X	NR	NR	NR	NR
Y*	NR	-		-

Presents the status (nonrandom or random) of CT organization for all chromosomes in resting (-PHA) and activated (+PHA) lymphocytes for each participant enrolled in this study. A minimum of 100 cells were captured and analyzed per CT, per subject in both resting (-PHA) and activated (+PHA) lymphocytes. The *χ* ^2^ goodness of fit test (d.f: 4) was utilized to determine whether the 2D radial distribution of each CT was random (*p* > 0.05) or nonrandom (*p* < 0.05). Nonrandom organization is denoted by NR (*p* < 0.05), random organization (*p* > 0.05) is denoted by blank boxes. *Data for the Y chromosome is only available from the male subject

### Spatial relationship between CTs within the 3D nucleus

**Fig. 2:**
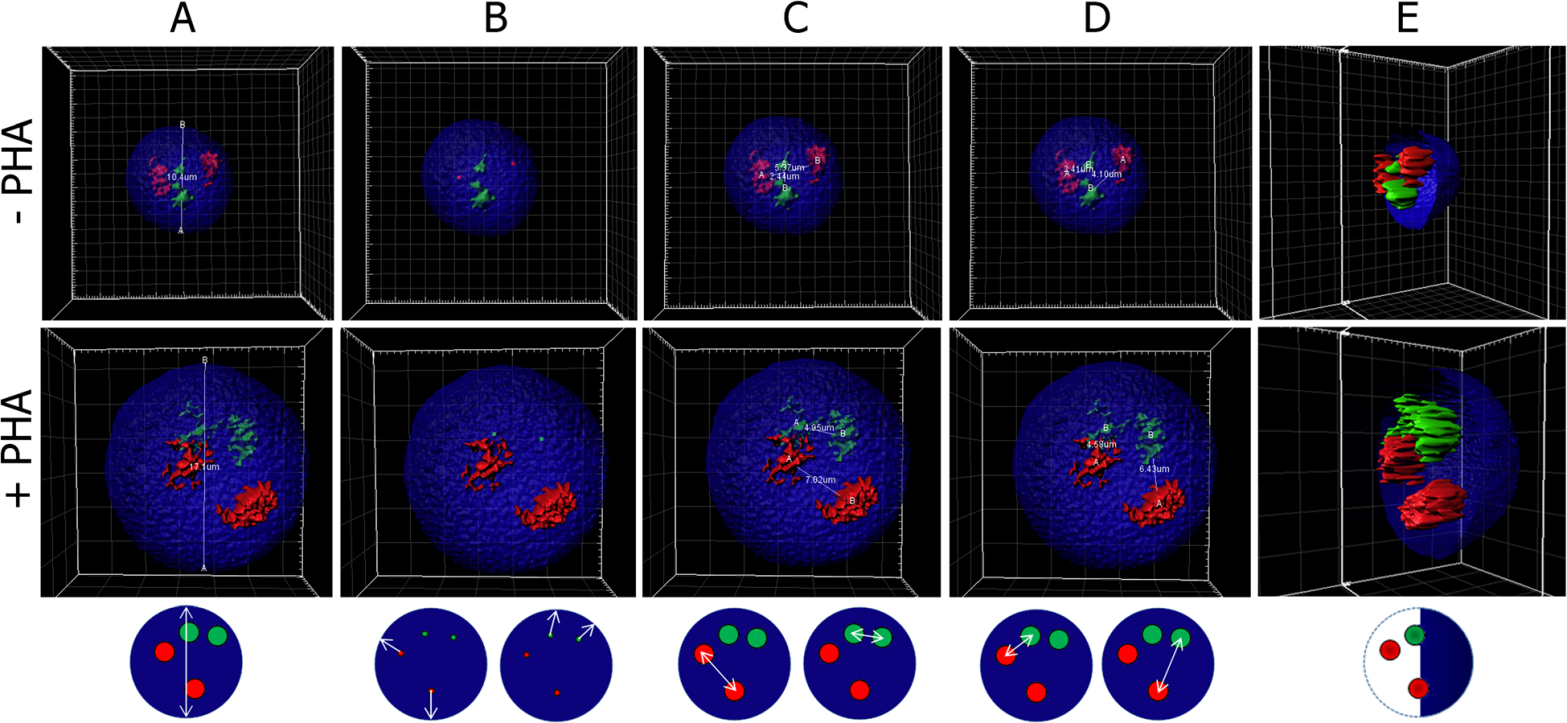
Examples of 3D models demonstrating the various methods of analysis. A representative image of 3D models and measurements performed using Imaris (V.7.6.3) is provided for CT 17 (red) and CT 22 (green) in a resting (-PHA) and activated (+PHA) lymphocyte nucleus with a schematic representation shown below. Panels **a-e**, each show the same same nucleus and provides an example of each measurement performed. Panel **a**: diameter of the nucleus (-PHA 10.4 μm; +PHA 17.1 μm). Panel **b**: geometrical center of each CT, which is utilized by the distance transformation (DT) as the point to measure to the nearest nuclear edge in any direction (shown for CT 17 -PHA; and CT 22 + PHA). DT measurements for each nucleus are: CT 17: 1.84 μm, 3.29 μm and CT 22 3.43 μm, 3.97 μm (-PHA), CT 17 2.68 μm, 3.23 μm and CT 22 1.5 μm 1.93 μm (+PHA). Panel **c**: homologous CT measurements between the center of each CT (CT 17 5.34 μm, CT 22 2.44 μm -PHA; and CT 17 7.02 μm, CT 22 4.95 μm + PHA). Panel **d**: heterologous CT measurements between the center of the closet heterologous CT pair (CT 17–22 2.41 μm -PHA; and CT 17–22 4.58 μm + PHA) and the furthest heterologous CT pair (CT 17–22 2.41 μm -PHA; and CT 17–22 4.58 μm + PHA). Panel **e**: the same cell that has been rotated toward the left with 50 % of the DAPI plane removed “clipped” to better demonstrate the 3D aspect of the CTs within a 3D nucleus

Dual color 3D FISH experiments utilizing the same CT pairs permits the spatial relationship between homologous CTs (intraprobe) and between heterologous CTs probed together (interprobe) to be investigated. 3D models were rendered in Imaris (V.7.6.3) and the intra- and interprobe measurements were established by measuring from the geometrical center of each CT between homologous CTs (intraprobe) and between heterologous CTs (interprobe) (Fig. 2). In addition, the 3D software also calculates the 3D volume of each rendered nucleus (μm^3^). A minimum of 40 cells per CT were studied (20 per subject), with exceptions for the sex chromosomes because: i) no intraprobe probe measurements are available for the Y chromosome; ii) intraprobe probe measurements for the X chromosome are available from the female subject only (20 cells); and iii) interprobe measurements between the X and Y chromosome are available from the male subject only (20 cells). In Table 2 we report these results: i) CT pairs probed together, ii) intra- and interprobe measurements (μm) for each CT, iii) nucleus diameter (μm); and iv) nucleus volume (μm^3^) in both resting and activated lymphocytes. The emerging picture from these data demonstrates that nuclei of activated lymphocytes were larger in size than that of resting lymphocytes (diameter and volume). In accordance with the increase in nucleus size, the intra- and inter-probe distances were also greater for all CTs in activated lymphocytes compared to resting lymphocytes. Prior to comparing the intra- and interprobe data in both resting and activated lymphocytes, the measurements obtained were normalized against the diameter of the nucleus to account for differences in nucleus size (data not shown). Utilizing these normalized values, it is possible to order CTs based on their proximity (closest - furthest). In resting lymphocytes the intraprobe measurements between homologous CTs (closest to furthest) were as follows: 19, 21, 22, 17, 1, 15, 16, 14, X, 10, 12, 6, 13, 20, 8, 11, 9, 18, 3, 7, 2, 4, and 5. In activated lymphocytes the distances between homologous CTs (closest to furthest) were: 22, X, 19, 21, 17, 12, 9, 14, 16, 18, 15, 3, 20, 8, 13, 4, 6, 10, 11, 5, 1, 2, and 7. When the relative distances are compared between resting and activated lymphocytes, 9 CTs demonstrated the largest increases in distances in activated lymphocytes (2, 11, 15, 19, 6, 7, 21, 10, and 1, respectively). Conversely, CT X in activated lymphocytes in the female subject showed a closer spatial organization to that seen in resting lymphocytes. The normalized interprobe distances among CTs when probed together in resting lymphocytes (closest to furthest) were: 17–22, 21–20, 14–16, 12–11, 8–7, X–Y, 15–3, 6–5, 10–9, 19–18, 1–13, and 2–4. In activated lymphocytes the spatial relationship between CT pairs (closest to furthest) were: 21–20, 12–11, 15–3, 19–18, 10–9, 14–16, 17–22, 6–5, 8–7, 1–13, 2–4 and X–Y. When comparing distances in activated lymphocytes compared to resting lymphocytes, five CT pairs demonstrated increasing distance (6–5, 14–16, 17–22, 8–7, and X–Y, respectively) whereas, 7 CT pairs demonstrated decreasing distance (1–13, 2–4, 15–3, 10–9, 21–20, 12–11, and 19–18, respectively).

**Table 2:** Spatial relationship of CTs, nuclear diameter, and volume within 3D resting and activated lymphocytes

CTs	PHA Status	Intraprobe left CT	Intraprobe right CT	Interprobe closest	Interprobe furthest	Nuclear diameter	Nuclear volume
		μm	μm	μm	μm	μm	μm^3^
1 – 13	-PHA	4.21 (1.07)	4.79 (1.28)	3.52 (1.75)	5.70 (2.0)	10.27 (0.98)	996.17 (278)
1 – 13	+PHA	10.32 (2.53)	8.82 (4.31)	5.48 (2.26)	9.59 (2.98)	17.15 (2.4)	2922.92 (1083)
2 – 4	-PHA	5.83 (2.06)	5.99 (1.66)	3.72 (1.57)	5.89 (1.87)	10.54 (0.88)	964.10 (137)
2 – 4	+PHA	10.42 (3.29)	8.99 (2.78)	5.01 (2.19)	9.76 (3.22)	16.65 (2.07)	3171.36 (1003)
15 – 3	-PHA	4.49 (1.11)	5.47 (1.95)	2.80 (1.42)	5.11 (1.87)	10.45 (1.02)	893.98 (236)
15 – 3	+PHA	8.62 (2.7)	8.71 (2.81)	4.00 (2.2)	8.48 (2.63)	17.23 (2.21)	3126.29 (1034)
6 – 5	-PHA	4.89 (1.64)	6.11 (2.03)	2.86 (1.28)	5.13 (1.59)	10.49 (1.11)	815.17 (238)
6 – 5	+PHA	8.74 (3.23)	9.48 (2.67)	4.31 (2.32)	8.80 (2.92)	15.93 (1.95)	2831.50 (849)
8 – 7	-PHA	5.36 (1.81)	5.99 (1.71)	2.92 (1.18)	5.49 (1.90)	11.18 (1.10)	971.98 (216)
8 – 7	+PHA	7.73 (3.47)	9.45 (2.84)	4.91 (2.66)	8.32 (2.58)	15.10 (2.25)	2422.48 (984)
10 – 9	-PHA	5.08 (1.82)	5.65 (2.03)	3.47 (1.59)	5.58 (1.78)	11.12 (0.88)	997.48 (227)
10 – 9	+PHA	8.79 (2.75)	7.48 (2.65)	4.31 (2.15)	7.99 (2.72)	15.89 (1.99)	2789.34 (936)
12 – 11	-PHA	4.87 (1.39)	5.17 (1.83)	2.81 (1.34)	5.05 (1.64)	10.62 (1.06)	806.81 (183)
12 – 11	+PHA	7.24 (2.67)	8.95 (3.14)	3.51 (2.12)	7.87 (3.39)	16.04 (2.41)	2628.15 (914)
14 – 16	-PHA	4.58 (1.52)	4.55 (1.62)	3.00 (1.59)	4.45 (1.48)	10.48 (0.99)	758.17 (181)
14 – 16	+PHA	7.83 (3.03)	7.86 (2.04)	4.50 (2.36)	8.29 (3.03)	16.40 (2.16)	2945.85 (1034)
17 – 22	-PHA	4.25 (1.52)	3.78 (1.29)	2.85 (1.06)	4.32 (1.66)	10.54 (1.07)	706.02 (173)
17 – 22	+PHA	5.88 (1.73)	4.81 (1.23)	3.95 (1.41)	6.41 (3.11)	13.14 (1.85)	2040.62 (757)
19 – 18	-PHA	3.26 (1.34)	5.38 (2.44)	3.40 (1.31)	5.36 (1.53)	10.48 (1.24)	873.05 (295)
19 – 18	+PHA	6.88 (1.71)	8.63 (3.77)	4.80 (2.66)	8.75 (3.02)	17.70 (2.3)	3333.78 (1151)
21 – 20	-PHA	3.60 (1.6)	5.17 (2.33)	2.39 (1.41)	5.12 (2.07)	10.98 (1.32)	931.65 (361)
21 – 20	+PHA	7.79 (3.2)	9.26 (3.42)	3.71 (2.03)	8.05 (3.72)	18.31 (2.61)	3530.47 (1335)
X – Y	-PHA	-	-	3.99 (1.72)	-	10.51 (1.07)	797.52 (224)
X – Y	+PHA	-	-	8.75 (3.69)	-	17.87 (1.84)	4225.90 (972)
X	-PHA	4.68 (1.83)	-	-	-	10.67 (0.95)	831.76 (197)
X	+PHA	5.32 (2.19)	-	-	-	10.76 (1.46)	1292.68 (480)

Provides an overview of all the 3D measurements obtained utilizing Imaris software (V7.6.3) for all 24 CTs in resting and activated lymphocytes. The CTs column indicates which CTs were probed together in the dual FISH experiment (e.g. 1 - 13 indicates chromosomes 1 and 13). PHA status indicates whether data is from resting (-PHA) or activated (+PHA) lymphocytes. The remaining columns present the average measurements in microns (μm or μm^3^) obtained from a minimum of 40 cells (20 per subject with the exception of the X–Y and X–X data, with 20 cells from the male and female subject evaluated, respectively). Fig. 2 provides examples of how each distance was measured. The standard deviation of the measurements in all studied cells is provided in parentheses. The intraprobe columns provides the measurement between homologous CTs, the first intraprobe column provides the distance between the first CT listed in the CT column (e.g. CT 1), with the second intraprobe column providing the distance between the homologous CT pair for the second CT in the CT column (e.g. CT 13). The two interprobe columns provides the distances between the two heterologous CTs (e.g. CT 1 and CT 13), the first interprobe column presents the distance for the two closest CTs, with the second column presenting the data for the two furthest CTs. Subsequently, both the nuclear diameter and volume is provided

### CT organization from the nuclear interior toward the nuclear periphery (2D and 3D approach)

Our 2D method of analysis allows the distribution of CT fluorescence to be transformed to provide a single number for each nucleus, which reflects the midpoint of the frequency distribution of observed fluorescence (median) across the five rings [20]. This median value can be utilized to establish the hierarchical radial order of CTs from the nuclear interior toward the nuclear periphery [20]. The midpoint of the frequency distribution of the fluorescence for each CT in resting and activated lymphocytes was determined from a minimum of 200 cells per CT (100 cells per subject), with the exception of the CT Y, (studied in 100 cells from the male subject) (Table 3). In addition, hierarchical radial organization of CTs in resting and activated lymphocytes was also established from the 3D data. The hierarchical order of all 24 CTs was determined by the distance in microns, measured from the geographical center of the CT to the nearest nuclear edge. A minimum of 40 cells per CT (20 cells per subject) were analyzed with the exception of CT Y. To account for differences in nucleus size, the measurements obtained were normalized against the radius of the nucleus (Table 3). Despite inherent differences in the 2D and 3D analysis (2D: CT median fluorescence intensity; and 3D: distance from the CT geometrical center to nearest nuclear edge) the hierarchical organization of CTs in resting lymphocytes is by and large very similar between the two methodologies (Table 3). Both 2D and 3D methods show the same CTs forming clusters that compose the core, intermediate and peripheral regions of the nucleus: i) CTs: 1, 15, 16, 17, 19, 21, 22; ii) CTs: 6, 8, 9, 10, 11, 12, 14, 20, X; and iii) CTs: 2, 3, 4, 5, 7, 13, 18 and Y (Table 3). The hierarchical organization of CTs in activated lymphocytes also demonstrates similar clustering; however, more variability in the clusters of CTs forming the core, intermediate and peripheral regions of the nucleus is observed between 2D and 3D methods (Table 3).

**Table 3:** Comparison of radial hierarchical organization of CTs between 2D and 3D methods

	CT hierarchy -PHA 2D (median)	CT hierarchy -PHA 3D (distance)	CT hierarchy + PHA 2D (median)	CT hierarchy + PHA 3D (distance)
Nuclear Interior	19 (1.78)	19 (0.61)	22 (1.99)	22 (0.52)
	17 (1.85)	22 (0.57)	17 (2.00)	19 (0.51)
	22 (1.85)	1 (0.50)	19 (2.06)	21 (0.48)
	1 (2.08)	15 (0.49)	21 (2.14)	17 (0.46)
	21 (2.10)	17 (0.49)	10 (2.27)	16 (0.42)
	16 (2.12)	16 (0.48)	15 (2.29)	1 (0.41)
	15 (2.17)	21 (0.46)	8 (2.31)	12 (0.41)
	9 (2.23)	20 (0.45)	14 (2.34)	15 (0.40)
	14 (2.25)	12 (0.44)	16 (2.34)	8 (0.40)
	20 (2.30)	14 (0.43)	12 (2.37)	10 (0.39)
	8 (2.33)	X (0.43)	6 (2.49)	7 (0.39)
	6 (2.34)	8 (0.42)	X (2.53)	20 (0.39)
	12 (2.34)	6 (0.42)	1 (2.54)	14 (0.39)
	10 (2.38)	11 (0.40)	20 (2.63)	6 (0.38)
	X (2.42)	10 (0.40)	4 (2.76)	9 (0.38)
	11 (2.49)	9 (0.39)	5 (2.85)	Y* (0.36)
	4 (2.53)	2 (0.37)	9 (2.89)	18 (0.35)
	7 (2.58)	Y* (0.37)	2 (2.96)	X (0.34)
	5 (2.62)	4 (0.37)	11 (2.96)	3 (0.34)
	2 (2.69)	7 (0.37)	Y* (2.98)	13 (0.33)
	13 (2.73)	13 (0.34)	3 (3.04)	5 (0.33)
	3 (2.77)	3 (0.34)	18 (3.04)	4 (0.32)
	18 (2.77)	18 (0.32)	13 (3.05)	2 (0.32)
Nuclear Periphery	Y* (2.83)	5 (0.32)	7 (3.12)	11 (0.31)

Presents the radial hierarchy for all 24 CTs in resting and activated lymphocytes using both 2D and 3D approaches from both subjects. CTs are ordered from the nuclear interior (top of the table) toward the nuclear periphery (bottom of the table). Following the CTs in parentheses are the numerical values used to order the CTs. In the 2D approach, CTs are ordered based on the midpoint of the distribution of the CT fluorescence across the five rings of equal area (median) (200 cells/CT). In the 3D approach, CTs are ordered based on their (distance) to the nearest nuclear edge following normalization against the radius of the nucleus to account for differences in nucleus size (40 cells/CT). Data for CT Y (Y*) is obtained from the male subject only (2D: 100 cells; 3D: 20 cells)

### CT repositioning following PHA activation of lymphocytes utilizing 2D and 3D approaches

Furthermore, we evaluated whether there was any statistically significant alteration in the localization of CTs between resting and activated lymphocytes within each of the subjects enrolled. To evaluate 2D CT repositioning, the percentage of fluorescence distribution in each shell (Fig. 1) was compared between resting and activated lymphocytes in each of the two subjects. Alteration were deemed statistically significant when the *p* value from the chi-squared goodness-of-fit comparison was less than 0.05. In total there were 15 significant CT repositioning events (10 in the male subject, and 5 in the female subject) from resting to activated lymphocytes (Table 4). Based on the histograms produced from the radial analysis, movement of CTs was classified into the following categories: 1) interior to less interior, 2) interior to intermediate, 3) interior to periphery, 4) intermediate to periphery, 5) intermediate to interior (Fig. 1, Table 4). CT 1 was the only CT that demonstrated statistically significant repositioning in activated lymphocytes in both subjects, and also depicted the same type of movement (interior to less interior). Interestingly, the only CT that demonstrated a significant repositioning event toward the nuclear interior was that of CT X in the male subject. Rather than assessing the radial distribution of the entire CT in a 2D object, 3D methodologies provides physical localization of the center of each CT in a 3D nucleus to the nearest nuclear edge in 3D models rendered in Imaris (V.7.6.3). As with the 2D analysis the nuclear localization of all 24 CTs was established in the same subjects in resting and activated lymphocytes. For the 3D analysis CT repositioning was established using a two-tailed, paired *t*-test (*p* < 0.05) to detect significant events of CT repositioning following activation of lymphocytes. In total 10 repositioning events were found to be statistically significant, six in the male subject (CTs 11, 14, 15, 16, 17, and 19) and four in the female subject (CTs 11, 16, 19, and X) (Table 4). All significant CT movements in activated lymphocytes were relatively more peripheral compared to resting lymphocytes. Three CTs were common between the two subjects (CTs 11, 16, and 19), whereas two CTs were common between the two methods in the male (CTs 14 and 16), and in the female (CTs 11 and 19), all demonstrating a similar peripheral movement.

## Discussion

The effects of transcriptional reprogramming on nuclear organization have been underexplored. Therefore the purpose of this study was to evaluate the consequences of PHA activation on global nuclear organization of CTs in human lymphocytes. To the best of our knowledge this is the first study to evaluate the radial organization, random/nonrandom status, intra- and interprobe spatial relationship between CTs, radial hierarchy and repositioning of all 24 chromosomes in resting and PHA-activated lymphocytes using both 2D and 3D approaches in multiple subjects.

**Table 4:** CTs exhibiting radial repositioning in activated lymphocytes compared to resting lymphocytes

2D repositioning			
Male Subject		Female Subject	
CTs	Movement	CTs	Movement
1, 6, 14, 16, 20	Less interior	1, 15, 19	Less interior
4, 5, 9	Interior to intermediate	11	Interior to intermediate
2	Intermediate to periphery	7	Interior to periphery
X	Intermediate to interior	-	-
3D repositioning			
Male Subject		Female Subject	
CTs	Movement	CTs	Movement
11, 14, 15, 16, 17, 19	More peripheral	11, 16, 19, X	More peripheral

CTs involved in statistical significant radial repositioning between resting and activated lymphocytes in both the male and female subject, as determined by 2D and 3D approaches. 2D radial repositioning was determined using the *χ* ^2^ goodness-of-fit test (*p* < 0.05). The direction of the repositioning movement was determined by comparing the radial distribution of CTs in each subject in resting and activated lymphocytes (Fig. 1). 3D radial repositioning was determined using the two-tailed, paired *t*-test (*p* < 0.05). The direction of the repositioning movement was determined based on the distance to the nuclear edge in activated lymphocytes compared to resting lymphocytes

The 2D radial organization of CTs demonstrates a highly reproducible radial CT organization with CTs displaying similar radial distributions and random/nonrandom organization between subjects in both resting and activated lymphocytes. Overall, the organization in activated lymphocytes appears to be more “relaxed” (or less defined), with more CTs (10 in the male subject and 7 in the female subject) displaying a random organization compared to resting lymphocytes (2 CTs in both subjects). It has been established that following PHA stimulation in human lymphocytes, there is a rapid transcriptional surge, which is accompanied by nuclear and nucleolar morphological changes [34]. Specifically, there is enlargement of the nuclear size, increased chromatin decondensation and activation of nucleoli [34]. In agreement with previously published studies, the current study also reports an increase in nuclear size (~2–3 fold) in activated lymphocytes compared to resting lymphocytes [34]. The increased nuclear size in activated lymphocytes could account for the more random CT organization observed. Chromatin is a dynamic entity in constant motion inside the nucleus [36, 37]; thus, in a nucleus with more available space, it is feasible that there is relatively more motion at least for some CTs, perhaps conferring a less defined organization than other CTs.

Dual color 3D FISH experiments also enabled the spatial relationship between CTs to be examined, providing information on interactions amongst homologous chromosomes and heterologous CT pairs. Differences in nuclear size is a potential confounder, therefore, to account for these differences the distances were normalized using the nuclear diameter. The distances between homologous CTs (intraprobe) were larger in 74 % (17 heterologous homologs) of cases in activated lymphocytes (CT Y excluded), with only 6 CTs possessing shorter distances. The interprobe results demonstrate that 58 % of CTs (14 CTs) had relatively smaller distances, with 42 % of CTs (10 CTs) possessing greater distances in activated lymphocytes compared to resting lymphocytes. In activated lymphocytes the following CT pairs demonstrated the largest increases in distance compared to resting lymphocytes: 17–22, 8–7 and X–Y (male subject only). Altered spatial organization between homologous CTs could be due to different patterns of chromatin decondensation (or condensation) amongst different CTs, or as result of the new transcriptional profile that occurs following PHA activation [34]. The spatial relationship between CTs may also be the result of the formation of new CT neighborhoods following transcriptional activation, or serve as a protective mechanism to reduce potential interactions to prevent any structural abnormalities (e.g. translocations) as result of DNA damage that increases during T lymphocyte activation [34, 38].

Utilizing both 2D and 3D methodologies the radial hierarchy of all CTs from the nuclear interior toward the nuclear periphery was established. The radial organization of CTs in both resting and activated lymphocytes in this study follows  a more gene-density correlation, which is in agreement with previously published literature [4, 11, 15, 20]. This type of organization might be stimulated by chromosome specific gene expression and the organization of transcription in the nucleus (i.e. interaction of polymerases with CT physical properties like NORs) [16, 39]. Both 2D and 3D approaches provided remarkably similar results in terms of the hierarchical order of CTs that preferentially formed clusters in the nuclear interior, intermediate and peripheral regions. Gene rich CTs (e.g. CTs 15, 17, 19, and 22) clustered toward the nuclear interior, whereas gene poor CTs (e.g. CTs 2, 3, 13, and 18) localized toward the nuclear periphery in resting and activated lymphocytes. CT 21 is the only gene poor chromosome associated with the nuclear interior [40], a finding mirrored in a recently published study [20]. The intermediate region of the nucleus is largely composed of the following CTs: 6, 8, 10, 12, 14, 20, and X, of these, four CTs were of intermediate gene density (CTs 6, 10, 14, and X), two were gene rich (CTs 12 and 20) and one was gene poor (CT 8) [40].

2D and 3D approaches were also utilized to evaluate any statistically significant events of CT repositioning between resting and activated lymphocytes. Both methods identified significant repositioning events with similarities emerging between the two approaches. These findings include more CTs repositioned in the male subject (e.g. 10 events, 2D and 6 events, 3D) compared to the female subject (5 events, 2D, and 4 events, 3D), and all CTs demonstrating a relatively more peripheral organization, with the exception of CT X (2D in the male subject). One repositioned CT (CT 1) was shared between the subjects in the 2D results with three repositioned CTs shared between the subjects using the 3D method (CTs 11, 16, and 19). When an intra-subject comparison was made between the two approaches (2D and 3D), CTs 14, and 16 and CTs 11 and 19 showed similar characteristics in the male and female subject, respectively. One possible explanation for the differences observed in the intra-subject comparisons regarding the number of CT repositioning events and the CTs involved could be due to variability in the nuclear size between the subjects. A two-tailed, paired *t*-test showed that in certain CTs (CTs 2, 4, 5, 6, 9, 10, 14, 16, 17, 22, X and Y) there was a significant difference (*p* < 0.05) in the nucleus size between the male and female subject activated lymphocytes (data not shown). Interestingly, 8 out of the 10 significant events of CT repositioning involved these CTs. A similar *t*-test in resting lymphocytes only revealed a significant difference in nucleus size between the two subjects for CTs 3 and 15 depicting more comparable nuclei sizes (data not shown). The differences in the radial positions could reflect the different network interactions of chromosomes with other nuclear structures or alternative territories [34]. To date, one other study has investigated the repositioning of 11 CTs (CTs 1, 2, 3, 4, 5, 9, 11, 12, 13, 21 and 22) in resting and activated lymphocytes [34]. This study, reported peripheral repositioning of CTs 1 and 3 and more internal repositioning of CT 21 [34]. The majority of repositioned CTs in the current study were not evaluated in the previous study, however, we confirm the peripheral repositioning of CT 1 but not for CT3, and although not significant in this study the radial hierarchy of CT 21 is more internally localized in activated lymphocytes.

An additional important aspect in the current study is the use of both 2D and 3D approaches to study CT organization. The two approaches to address CT organization are synergistic, the 2D approach assesses the radial distribution of the entire CT in a 2D nucleus, whereas with the 3D approach assesses the physical localization of the center of the CT to the nearest nuclear edge or another CT. Despite the inherent differences between the two approaches the relatively small variations observed in the hierarchical organization of CTs are reassuring, suggesting reproducibility between both 2D and 3D methods. The reproducibility between the two approaches is likely due to the fact that the 2D data was transformed to a single data point, similar to the 3D data, rather than examining the distribution of the entire CT. Greater variability was observed between the two approaches when examining CT repositioning in activated lymphocytes, most likely reflecting differences when studying localization of the entire CT (2D) versus a single point (3D). Nevertheless, some CTs demonstrated reproducible repositioning using both methods which provides further evidence that both 2D and 3D approaches provide comparable data [32].

## Conclusions

Alterations observed in CT organization in activated lymphocytes most likely depend on and influence the spatial organization of CTs [34]. Both 2D and 3D approaches revealed CT specific differences in organization between resting and activated lymphocytes. These differences were associated with the increased nuclear size and altered chromatin condensation observed in activated cells, and may be related to the altered transcriptional profile in activated cells. Different CT interactions (changes in intra- and interprobe spatial organization) may lead to the formation of different CT neighborhoods. Furthermore, there is evidence to indicate that CTs in activated cells display less intermingling which may serve as a mechanism to reduce the formation of chromosome aberrations [34]. These hypotheses warrant further investigation in future studies focusing on the spatial relationship and interaction between CTs. In addition, future studies should include a larger cohort and genome-wide gene expression studies to determine whether consistency is observed among individuals for CT repositioning events and whether repositioning is associated with gene expression.

## Methods

### Sample cohort

This research study was approved by the Florida International University Institutional Review Board (protocol numbers: IRB-121010-00, IRB-14-0163). Informed consent was provided by two individuals (one male and one female subject) to participate in this study. Both participants were 35-years-old, karyotypically normal (data not shown) and completed a brief health history survey, providing life style (e.g. alcohol or tobacco use) any recent illness information, and any medication taken. Both participants were non-smokers and had not knowingly been in contact with any hazardous or radioactive material in their working or home environment and were occasional social drinkers (2–8 units/week).

### Conditions of Cell Culture

Peripheral blood was collected by venipuncture in heparin tubes (Greiner-BioOne, Monroe, NC, USA). Whole blood from each individual was split and cultured in the presence or absence of PHA. All lymphocyte cultures were prepared in RPMI 1640 (Lonza, Walkersville, MD, USA) reconstituted with: 10 % heat inactivated fetal bovine serum (FBS – Sigma-Aldrich, St Louis, MO, USA), 2 % L-glutamine (Thermo-Fisher, Waltham, MA, USA) and 1 % penicillin-streptomycin solution (Thermo-Fisher, Waltham, MA, USA). All cultures had a total volume of 5 ml and for those that PHA was added, they were reconstituted with 100 μl of PHA, (45 mg/vial) (Remel Inc, Lenexa, KS, USA) 0.8–1.0 ml of blood was incubated for 71 h at 37 °C (5 % CO_2_). Following lymphocyte culture incubation (71 h), lymphocytes were prepared following standard karyotyping protocols. Proliferating cells in metaphase were arrested using 0.2 μg colcemid (Thermo-Fisher, Waltham, MA, USA) for 30 min at 37 °C, followed by standard hypotonic conditions to allow separation of white blood cells from anucleate erythrocytes (0.075 M of KCL - Thermo-Fisher, Waltham, MA, USA) for 45 min at 37 °C. White blood cells were subsequently fixed in 3:1 (v/v) of methanol:acetic acid solution to clean and fix the preparation. All cultures were stored at -20 °C immediately following the harvesting procedure.

### Fluorescence in situ hybridization (FISH)

Cells cultured in the presence and absence of PHA were dropped on glass slides (FisherBrand® - Thermo-Fisher, Waltham, MA, USA), allowed to adhere by ageing overnight at room temperature (RT) and subsequently washed in 1X PBS (Thermo-Fisher, Waltham, MA, USA), followed by an ethanol dehydration step (70–80-100 %, 3 min each). Air dried cells were treated with a 1 % pepsin solution (Thermo-Fisher, Waltham, MA, USA) in a pre-warmed at 37 °C solution of 49 ml double distilled water (ddH_2_O) and 0.5 ml of 1 N HCL (Thermo-Fisher, Waltham, MA, USA) for 20 min. Cells were rinsed with ddH_2_O and 1 X PBS at RT, and subjected to another round of fixation using 1 % paraformaldehyde/PBS (Thermo-Fisher, Waltham, MA, USA) at 4 °C for 10 min. Following which, slides were rinsed in 1 X PBS and ddH_2_O (RT), prior to another ethanol dehydration series (2 min each), and finally air dried. A dual color FISH experiment (fluorescein isothiocyanate (FITC), and tetramethyl rhodamine isothiocyanate (TRITC) labelled FISH probes) was performed utilizing whole chromosome paints (WCPs), for all 24 chromosomes (Rainbow Scientific, Windsor, CT, USA). WCPs were co-denatured for 5 min with lymphocytes at 75 °C followed by overnight hybridization (>16 h) at 37 °C using a Thermobrite® Statspin (Abbott Molecular, Illinois, IL, USA). A post hybridization stringency wash was performed in a pre-warmed 73 °C solution of 0.7 X SSC/0.3 % Tween 20 (Thermo-Fisher, Waltham, MA, USA) (35 ml of 20 X SSC, 3 ml of Tween 20 and 965 ml of ddH_2_O) for 2 min. After 2 min elapsed, cells were washed in 2 X SSC/ 0.1 % Tween 20 (100 ml of 20 X SSC followed by a brief ethanol series (1 min each). Slides were subsequently air dried and mounted with 4′,6-diamidino-2-phenylindole (DAPI – Vector Labs, Burlingame, CA, USA) under a 24X55mm coverslip.

### 2D Image acquisition, radial chromosome positioning and statistical analysis

All images for 2D analysis were captured using an Olympus BX61 epifluorescence microscope equipped with a cool charged couple device camera (Hamamatsu ORCA – R_2_ C10600) and a motorized ES111 Optiscan stage (Prior Scientific UK). Three single band pass filters for FITC, TRITC, and DAPI (Chroma Technology, Bellows Falls, VT, USA) were used. All images were acquired using Smart Capture 3.0 (Digital Scientific, UK) exported as .tiff files for further analysis. A minimum of 100 cells were analyzed per subject, per chromosome pair, per condition (-PHA and + PHA). To evaluate the radial chromosome position previously published methodologies were utilized [11, 20]. The details have been described extensively elsewhere [20, 26, 28]. In brief, a customized script written for Image J allows the separation of each captured image into three channels (FITC, TRITC and DAPI counterstain). The DAPI fluorescence is converted into a binary mask that allows for the creation of 5 concentric rings of equal area from nuclear interior toward the nuclear periphery. The proportion of WCP signal in each ring, for each channel (FITC and TRITC) is subsequently measured relative to the total signal for the area contained within the ring. Data was normalized against the different DNA content in the nucleus to compensate for the fact that a 3D object is observed in 2D. The Chi-squared goodness of fit (*χ*^2^) was utilized to evaluate if the organization of each chromosome differed from random (*p* < 0.05) and to compare differences between conditions (-PHA and + PHA) in each subject.

### 3D Image acquisition, chromosome territory positioning and statistical analysis

The FISH protocol described above was used to prepare slides for 3D image acquisition. The images were captured using a DeltaVision (Applied Precision, WA, USA) imaging station consisting of an Olympus IX71 inverted microscope with x 100, 1.4 NA oil-immersion lens and a photometric CCD. All images were taken with a Z step size of 1.0 μm (36 optical sections), saved as 3D stacks and subjected to constrained iterative deconvolution (using DeltaVision – SoftWoRx -version 5.5 - software package - Applied Precision, WA, USA). A TRITC (594 nm), FITC (523 nm) and DAPI (435 nm) filter were used to capture the images and a minimum 20 images per CT, per subject, per condition (-PHA and + PHA) were captured. Images were exported as 3D stacks to Imaris software (V.7.6.3 Bitplane – Zurich, Switzerland) to perform 3D model reconstruction for each of the captured images and further analysis. The embedded distance transformation (DT) tool was used to calculate the distance from the center of the CT (geometric center) to the nearest nuclear edge of the reconstructed 3D model image. In addition, distances from the center of each CT were measured between homologous CTs (intraprobe) and heterologous CT pairs (interprobe). The measurements were normalized against the radius of the nucleus to account for differences in the sizes of each nuclei and the nucleus volume was calculated in Imaris. A two-tailed paired *t*-test (*p* < 0.05) was used to evaluate any statistical significant differences in the repositioning of CTs (-PHA and + PHA) and to evaluate significant differences between intra- and interprobe distances and the volume of the nuclei.

## Abbreviations

2D: 2 dimensions
3D: 3 dimensions
CCD: Charge Couple Device
CT(s): Chromosome Territory (ies)
DAPI: 4′,6-diamidino-2-phenylindole
ddH_2_O: Double distilled water
DT: Distance transformation
FISH: Fluorescent in situ hybridization
FITC: Fluorescein isothiocyanate
PBS: Phosphate buffer saline
PHA: Phytohaemagglutinin
RT: Room temperature
SSC: Saline sodium citrate
WCP: Whole chromosome paint
